# Diagnosis of Two Unrelated Syndromes of Prader-Willi and Calpainopathy: Insight from Trio Whole Genome Analysis and Isodisomy Mapping

**DOI:** 10.3390/genes15070946

**Published:** 2024-07-19

**Authors:** Mario Cuk, Busra Unal, Andjela Bevanda, Connor P. Hayes, McKenzie Walker, Feruza Abraamyan, Robert Beluzic, Kristina Crkvenac Gornik, David Ozretic, Maja Prutki, Qian Nie, Honey V. Reddi, Arezou A. Ghazani

**Affiliations:** 1 Department of Pediatrics, School of Medicine, University Hospital Centre Zagreb, 10000 Zagreb, Croatia; mcuk@kbc-zagreb.hr; 2 Division of Genetics, Brigham and Women’s Hospital, Boston, MA 02115, USA; busraunalbav@gmail.com (B.U.); connorhayes20@yahoo.com (C.P.H.); mwalker46@bwh.harvard.edu (M.W.); fabraamyan@gmail.com (F.A.); 3 Zagreb County Health Center, 10000 Zagreb, Croatia; andjela.bevanda@gmail.com; 4 Division of Molecular Medicine, Rudjer Boskovic Institute, 10000 Zagreb, Croatia; robert.beluzic@gmail.com; 5 Department of Laboratory Diagnostics, Division of Cytogenetics, University Hospital Centre Zagreb, 10000 Zagreb, Croatia; kristina.crkvenac@kbc-zagreb.hr; 6 Department for Diagnostic and Interventional Neuroradiology, University Hospital Centre Zagreb, 10000 Zagreb, Croatia; david.ozretic@kbc-zagreb.hr; 7 Department of Radiology, School of Medicine, University Hospital Center Zagreb, 10000 Zagreb, Croatia; maja.prutki@gmail.com; 8 Precision Medicine Laboratory, Medical College of Wisconsin, Milwaukee, WI 53226, USA; qnie@mcw.edu (Q.N.); hreddi@mcw.edu (H.V.R.); 9 Department of Medicine, Brigham and Women’s Hospital, Boston, MA 02115, USA; 10 Harvard Medical School, Boston, MA 02115, USA

**Keywords:** joint WGS analysis, uniparental disomy, isodisomy, heterodisomy, Prader-Willi syndrome, calpainopathy

## Abstract

Purpose: An investigation for the co-occurrence of two unrelated genetic disorders of muscular dystrophy and Prader-Willi syndrome (PWS) (OMIM#176270) using joint whole genome sequencing (WGS). Methods: Trio WGS joint analysis was performed to investigate the genetic etiology in a proband with PWS, prolonged muscular hypotonia associated hyperCKemia, and early-onset obesity. The parents were unaffected. Results: Results showed maternal isodisomy uniparental disomy (UPD) in chromosome 15, expanding from 15q11.2 to 15q22.2, including PWS regions at 15q11.2–15q13. Maternal heterodisomy was detected from 15q22.2 to 15q26.3. A pathogenic variant, NM_000070.3(CAPN3):c.550del (p.Thr184fs), was identified at 15q15.1 in a heterozygous state in the mother that was homozygous in the proband due to maternal isodisomy. Conclusion: This is the first study of the concurrent molecular etiology of PWS and calpainopathy (OMIM#253600) in the same patient. This report highlights the utility of joint analysis and the need for the assessment of autosomal recessive disease in regions of isodisomy in patients with complex and unexplained phenotypes.

## 1. Introduction

Joint analyses of WGS data enable the investigation of phasing and uniparental disomy (UPD) in complex genetic diseases [1,2]. Uniparental disomy (UPD) events may cause additional genetic abnormalities by unmasking deleterious recessive alleles inherited from the parent from whom UPD originated. Indeed, the co-occurrence of unrelated disorders such as Charcot-Marie-Tooth, Gaucher disease type 3, cystinuria, Crigler-Najjar syndrome type I, and long-chain 3-hydroxy acyl-CoA dehydrogenase deficiency has been reported [3,4,5].

Herein, we describe the investigation and molecular diagnosis of two unrelated genetic syndromes in a patient with the complex phenotype of PWS and calpainopathy due to maternal isodisomy. Prader-Willi syndrome (PWS) is a rare neurodevelopmental disorder with an estimated incidence in 1:10,000–1:30,000 live births. Maternal UPD is the second most common molecular etiology of PWS, resulting in a paternal expression loss of imprinting in the 15q11–q13 locus [6,7,8]. While chromosome 15 is well known for its association with PWS, it encompasses many other genes associated with autosomal recessive conditions. One such gene is *CAPN3*, known to cause calpainopathy [9], an autosomal recessive muscular dystrophy. With a prevalence of 1:100,000 [10], calpainopathy (MIM#253600) is the most common muscular dystrophy subtype, accounting for 30% of limb-girdle muscle dystrophies [11,12,13]. As a single gene disorder, the phenotypical features of calpainopathy consist of the symmetric and progressive weakness of proximal limb-girdle muscles, tiptoe walking, difficulty in running, scapular winging, a waddling gait, laxity of the abdominal muscles, Achilles tendon shortening, and scoliosis. Generally, cardiac muscles are not affected [14].

This is the first report of PWS and calpainopathy co-occurrence in the same individual due to isodisomy. Given the frequency of pathogenic variants in *CAPN3*, this report highlights the significance of extended joint genome analysis in PWS patients with complex phenotypes.

## 2. Participants, Materials, and Methods

### 2.1. Participants

The index patient, a two-year-old female, and her unaffected mother and father have consented to the study at the Department of Pediatrics, University Hospital Centre Zagreb, and enrolled in the CROseq genome program. CROseq Genome Program is a collaborative research program between Brigham and Women’s Hospital (BWH) (Boston, MA, USA), and the Department of Pediatrics, University Hospital Centre Zagreb (Zagreb, Croatia), supported by the Mila Za Sve Foundation (Rijeka, Croatia). Participants consented to the study at the University Hospital Center in Zagreb.

### 2.2. Karyotyping

Conventional karyotyping was performed to detect chromosomal abnormalities. The lymphocytes were derived from the peripheral blood. The cultured lymphocytes were treated with 10 μL/mL colcemid when they reached the logarithmic phase. Then, the fixative was added to preserve the structure of the metaphase cells. G-banding was applied to the harvested chromosomes by using Trypsin and Giemsa. The chromosomes’ morphology and number were evaluated through 20 metaphases. The karyotype result was described per The International System for Human Cytogenomic Nomenclature (ISCN) 2020.

### 2.3. Methylation Specific-Multiplex Ligation-Dependent Probe Amplification

Genomic DNA was purified from whole peripheral blood by FlexiGene DNA Kit according to the manufacturer’s protocol (Qiagen, Hilden, Germany). ME028-C1 kit was applied for MS-MLPA (MRC-Holland, Amsterdam, The Netherlands). The probemix of ME028-C1 contains probes specific for the PWS region and probes targeting sequences restricted by methylation-sensitive HhaI endonuclease. The latter probes were responsible for determining the methylation profiling. In addition, there were reference probes for genes located outside the PWS region. Internal control probe normalization was used to normalize peak intensities, and the intensity ratios of identical probes from the sample were compared with the reference. The amplified products were quantified by ABI 3130xl capillary sequencer (Applied Biosystems, Foster City, CA, USA). The final data were analyzed with Coffalyser software v2 (MRC-Holland, Amsterdam, The Netherlands). If there were copy number variation (CNV), the methylation pattern would be used to determine the CNV’s parental origin. However, in the absence of CNV, the methylation profile differentiated biparental inheritance from UPD.

### 2.4. Whole Genome Sequencing

In total, 2 mL of whole blood samples per participant were collected in an EDTA tube at the University Hospital Center Zagreb, Croatia. Genomic DNA purification and WGS were carried out at the Medical College of Wisconsin (Milwaukee, WI, USA). Genomic DNA with a purity ratio of 1.75–2.0 was obtained from the whole peripheral blood sample. Following the robotic library preparation, the Illumina NovaSeq 6000 platform (Illumina, CA, USA), with an average depth of coverage of 30×, was used for sequencing.

### 2.5. Zygosity and UPD Landscape Analysis by Trio WGS

To investigate maternal UPD in chromosome 15, the maternal and paternal contribution was assessed by joint trio WGS. All protein-coding genes over chromosome 15 were queried. The zygosity of the variants over the clinically relevant genes, as defined in OMIM, was further analyzed. To exclude the common variants that can potentially present in the father due to high population allelic frequency, the 1% allele frequency filter was applied. UPD landscape analysis by joint trio WGS was conducted using a single nucleotide polymorphism analysis of both the parents and the patient. The variants homozygous in the proband, heterozygous or homozygous in the relevant parent, and wild type in the other parent are labeled as UPD supportive variants. Supportive variants were clustered. Clusters of at least 4 Mbps with a minimum of 20 supporting variants were used to locate potential UPD regions. The parent of origin was assigned by the inheritance patterns located within each UPD region. UPD regions were attributed as maternal if the only contributor in the proband was the maternal allele.

### 2.6. UPD and Region of Homozygosity (ROH) Mapping

The regions of homozygosity (ROH) were used to further analyze the UPD region. Bcftools ROH module was used to assess the distribution and size of ROH. GVCF files of proband, mother, and father were merged with GATK CombineGVFs. Then, this file was processed using GATKGenotypeGVCFs. The resulting multi-sample VCF file was evaluated through UPDio (https://github.com/findingdan/UPDio, accessed on 2 February 2024). The regions of homozygosity (ROH) were graphically visualized by the variant caller that detects and outputs the runs of homozygosity from whole genome calls on autosomal human chromosomes.

### 2.7. Focused Trio WGS Analysis on Muscular Dystrophy

A trio-based whole genome analysis was performed due to the proband’s elevated creatinine kinase levels, muscular dystrophy, and early-onset obesity without hyperphagia. A focused interrogation was performed on the variants across the genes in chromosome 15. A technical assessment of variants was conducted to include only high- and medium-confidence variants. Low-confidence and failed variants were excluded from the analysis. Using Human Phenotype Ontology (HPO) terms for the probands’ phenotypes, variants were prioritized according to the gene’s association with these HPOs. The following HPOs were used: HP:0001263 Global developmental delay, HP:0001319 Neonatal hypotonia, HP:0003741 Congenital muscular dystrophy. Variants were subsequently filtered based on genomic region to exclude intronic, intergenic, and UTR variants. Both compound heterozygous and *de novo* variant sets were assessed separately.

### 2.8. Genome Variant Classification

The variant interpretation was performed based on the ACMG-AMP guideline [15]. The population allele frequency of the variants was evaluated with gnomAD (v2.1.1) aggregated allele frequency. Aggregated scores from the in silico prediction tools, including Polyphen, SIFT, MutationTaster, Mutation Assessor, FATHMM, FITCONS, GENOCANYON, dbscSNV ADA, and dbscSNV RF, were used to predict the effects of missense variants. A score of 0.7 was assigned for the deleterious impact, and scores less than 0.15 were used as a benign threshold. For the splice variants, SpliceAI with the upper and lower thresholds of 0.7 and 0.2, respectively, was used. Previous submissions in ClinVar were considered in the variant assessment. Additionally, online databases such as PubMed, OMIM, Orphanet, and GeneReviews were utilized to evaluate gene-phenotype association.

## 3. Results

### 3.1. Clinical Findings

**Figure 1:**
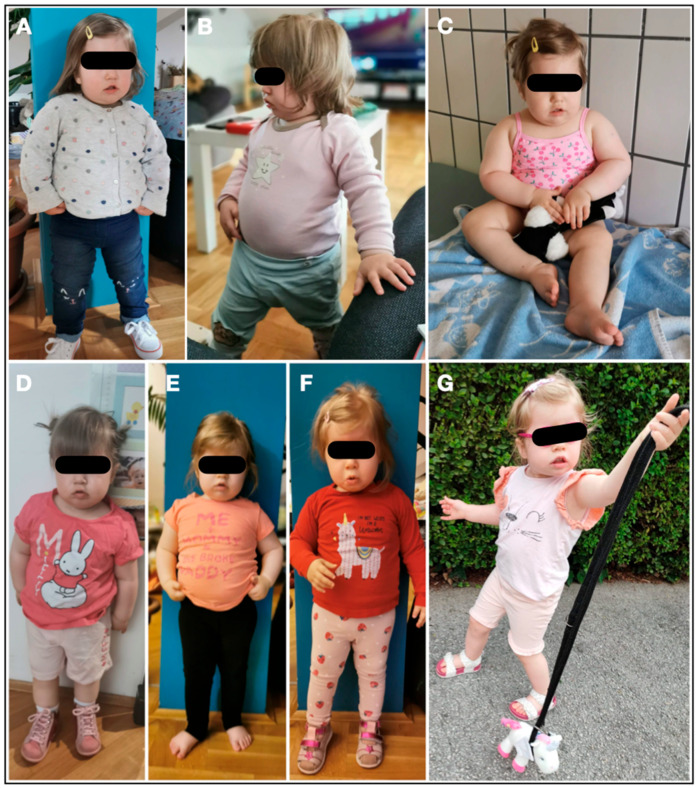
Initial clinical presentation of the patient and transforming effect of therapy from (**A**–**G**). Collectively, photographs are shown according to the age of the patient, from age 19 to 35 months and corresponding nutritional phases 1b and 2a. (**A**–**C**) Early-onset, non-hyperphagic obesity in the proband on at age of 19 months (Body weight: 13 kg, body height: 79 cm, BMI: 20.8 kg/m^2^). (**D**) At age 21 months, growth hormone, diet, and physical habilitation therapy were introduced. After introducing growth hormone, diet, and physical habilitation therapy, BMI progressively decreased at the age of 21 months (**D**), 24 months (**E**), and 30 months (**F**) and 35 months of age (**G**) and nutritional phase 2a (14 months after therapy introduction).

A two-year-old female patient has been admitted to the Department of Pediatrics, University Hospital Centre Zagreb, due to neurodevelopmental delay, congenital muscular dystrophy, severe central early-onset obesity without hyperphagia, distinctive facial features, and a prenatal history of intrauterine growth restriction (IUGR), oligohydramnios, and decreased fetal movements (Figure 1). The postnatal physical findings of birth weight (2970 g), length (48 cm), and head circumference (32.5 cm), all below the 3rd percentile, were compliant with intrauterine growth restriction. In the physical examination, metatarsus varus and equinovarus were inspected on the right and left foot, respectively. Characteristic dysmorphic and clinical features consistent with PWS were detected.

**Figure 2:**
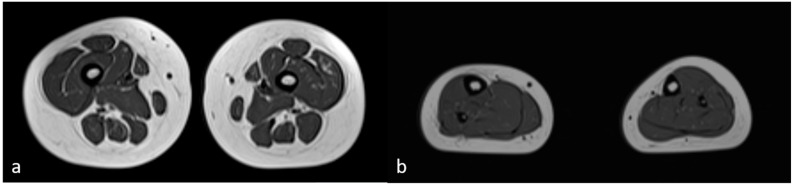
Magnetic resonance imaging of skeletal muscles (MRI). Axial T1-weighted images of proximal right and left (**a**) and distal right and left (**b**) muscle groups of the lower extremities showed minimal signs of fatty replacement of quadriceps muscles and relative sparing of muscles belonging to the distal muscle groups.

The neuromuscular abnormalities were present shortly after birth as determined by weak cry and poor suck, severe central generalized muscular hypotonia, and absence of deep tendon reflexes. Over the years, the motor milestones were delayed (unsupported sitting at 10 months, standing with support at 22 months, and walking at 30 months). The lack of improvement in hypotonia and deep tendon reflexes was unusual for PWS. Serum creatinine kinase (CK) level was elevated to 5×, a finding that is not consistent with PWS. Generalized tonic–clonic epileptic seizures with loss of consciousness presented at 3 years of age. CK level continuously increased and was elevated to 10×. Magnetic resonance imaging (MRI) of skeletal proximal and distal muscle groups of the lower extremities showed signs of muscle wasting in the proximal extremities and the relative sparing of muscles in the distal muscle groups (Figure 2).

Additionally, unlike typical PWS, severe central obesity was prominent at 19 months beyond the PWS’s hyperphagic nutritional phase 2b onset (BW 13 kg, BMI 20.8 kg/m^2^) (Figure 1). Both body weight for height and BMI had an early onset and rapid gain, which was unusual for PWS, particularly with the absence of hyperphagia in the proband.

### 3.2. Genomic Findings

#### Karyotyping and Methylation Analysis

The G-banded karyotype analysis was unremarkable, showing a 46,XX finding. The MS-MLPA analysis showed an aberrant methylation profile in the PWS region. It determined a hypermethylation pattern in *SNRPN* and *MAGEL* genes. The methylation level was 100% in these genes. No copy number variations (CNVs) were identified (Supplementary Figure S1). These results were consistent with PWS diagnosis. MS-MLPA could not delineate the causative molecular events for PWS, including possible imprinting defects by epimutation or UPD. Additionally, the absence of deep tendon reflexes, prolonged hypotonia, and elevated CK could not be explained by this finding. A comprehensive joint WGS was performed.

### 3.3. Ascertainment of UPD by Trio-WGS

The joint analysis of the PWS critical region in 15q11.2–q13 (MKRN3, MAGEL2, NDN, PWRN1, NPAP1, SNURF, SNRNP, SNORD107, SNORD64, SNORD108, SNORD109A, SNORD116, IPW, SNORD115, SNORD109B, ATP10A, GABRB3, GABRA5, GABRG3, OCA2, HERC2, APBA2, CHRNA7) showed maternal-only contribution in the proband. Variants in these regions were heterozygous in the mother and homozygous in the proband.

**Figure 3:**
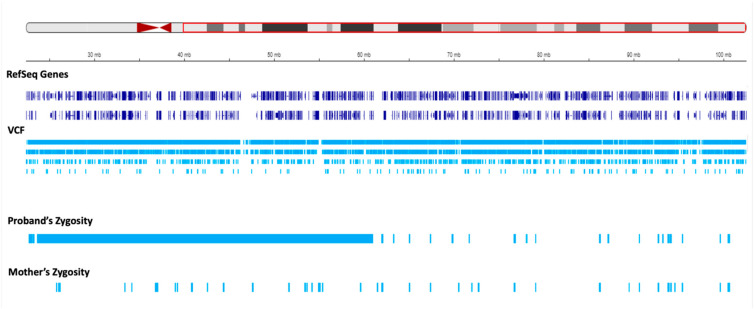
Regions of homozygosity (ROH) in chromosome 15q using joint whole genome sequencing (WGS) data. The region in the ideogram in the red box corresponds to chr15: g.22373341-g.102531392. The tracks below the ideogram show the analyzed data corresponding to the region in the red box. The reference sequence (RefSeq) and variant call format (VCF) tracks are depicted below. The proband and mother’s zygosity tracks show the region of homozygosity (ROH) in the proband. The proband‘s ROH is continuous within the maternal isodisomy contributed segment.

**Figure 4:**
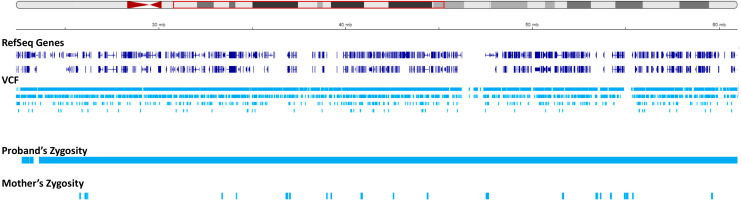
Status of zygosity in the chr15: 22373341-60984740 region. The red region in the ideogram shows the analyzed region. The tracks show the data corresponding to the region in the red box in the ideogram. The reference sequence (RefSeq) and variant call format (VCF) tracks are depicted below. The proband and mother’s zygosity tracks show homozygosity and heterozygosity status, respectively, resulting in segmental isodisomy in the proband.

**Figure 5:**
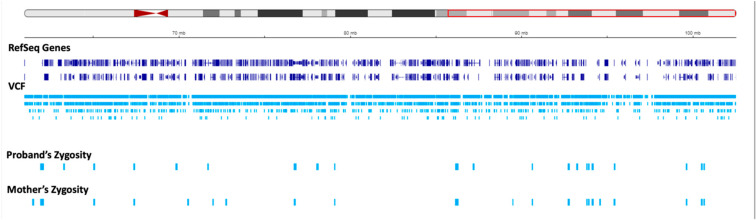
Status of zygosity in the chr15: 60984741-102531392 region. The red region in the ideogram shows the analyzed region. The tracks show the data corresponding to the region in the red box in the ideogram. The reference sequence (RefSeq) and variant call format (VCF) tracks are depicted below. The proband and mother’s zygosity tracks both show heterozygosity status in this region, corresponding to the segmental heterodisomy in the proband.

We set to investigate the extent of maternal contribution in 15q by performing a global UPD-ROH mapping in 15q to delineate the boundaries of isodisomy. In the proband, a continuous ROH was identified on chromosome 15 from 15q11.2 to 15q22.2 (Figure 3 and Figure 4). The isodisomy, consisting of 38.6 Mb in this region, was shown by the complete mapping of ROH with UPD with only maternal contribution (Table 1 and Figure 4). Telomeric to 15q22.2, the ROH was detected (Figure 5). The UPD-ROH mapping of this 41.55 Mb region was consistent with heterodisomy, and the inheritance pattern showed this region was also maternal only (Table 1). Variant level zygosity is shown for 25,091 variants of a selected 270 OMIM genes within 15q that included the PWS region of 15q11.2–q13 (Supplementary Table S1). An allele frequency filter of 1% was used to exclude common variants.

### 3.4. Muscular Dystrophy Assessment Using Trio WGS Analysis

**Figure 6:**
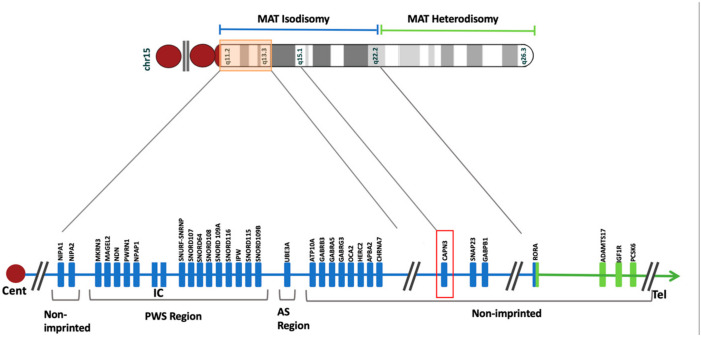
Ideogram of chromosome 15 and the schematic map of maternal uniparental disomy. The maternal isodisomy and heterodisomy contributions are coded with blue and green colors, respectively. The orange rectangular box highlights critical regions for Angelman Syndrome (AS) and Prader-Willi Syndrome (PWS). The imprinting center (IC) is located in this region. In the isodisomy contribution, *CAPN3* is marked in red. The breakpoint between isodisomy and heterodisomy occurs at *RORA*. The blue box illustrates the part of *RORA* within the maternal isodisomy region, and the green one shows the region with maternal heterodisomy. The template ideogram has been generated in R version 4.2.2 with GenomicRanges and ggbio packages [18,19] based on UCSC hg 19.

**Figure 7:**
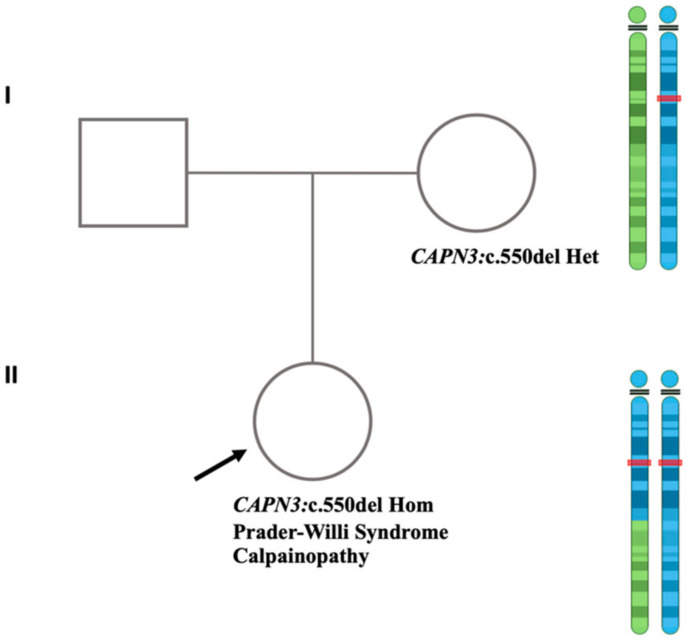
The pedigree of the family, schematically demonstrating the homodisomy of the maternal NM_000070.3(CAPN3):c.550del (p.Thr184fs) variant in the proband as the result of the maternal isodisomy event. Circles and squares represent female and male family members, respectively. The arrow indicates the proband diagnosed with Prader-Willi syndrome and Calpainopathy. The blue and green ideograms next to the mother in the pedigree represent the two different chromosomes 15 in the mother. The blue and green areas in the ideograms next to the proband in the pedigree denote the maternally inherited regions in chromosome 15 that are present in the proband. The red mark highlights the location of the *CAPN3*:c.550del variant.

A separate joint analysis for congenital muscular dystrophy (HP:0003741) was performed. This analysis revealed a pathogenic variant in the *CAPN3* (HGNC:1480) gene that is associated with calpainopathy, a rare limb-girdle muscular dystrophy (MIM#253600). The NM_000070.3(CAPN3):c.550del (p.Thr184fs) variant is located in the 15q15.1 isodisomy region (Figure 6), and was heterozygous in the mother and homozygous in the proband. The *CAPN3:*c.550del (p.Thr184fs) variant is a pathogenic frameshift variant (ClinVar ID: 17621) and has been reported in autosomal recessive calpainopathy patients [16]. The *CAPN3* gene constraint score of o/e and the pLI of *CAPN3* are 0.965 (0.56–0.96) and 0, respectively. The population allele frequency for this variant in the gnomAD database has been reported as 0.00023. *CAPN3*:c.550del is the most frequent pathogenic *CAPN3* variant in countries such as Croatia, Russia, Turkey, and Germany [17]. The isodisomy in the 15q15.1 region resulted in the isozygosity of the *CAPN3:*c.550del (p.Thr184fs) variant in the proband, which in turn led to the diagnosis of calpainopathy (Figure 7).

### 3.5. Assessment of the Breakpoint within UPD Region

**Figure 8:**
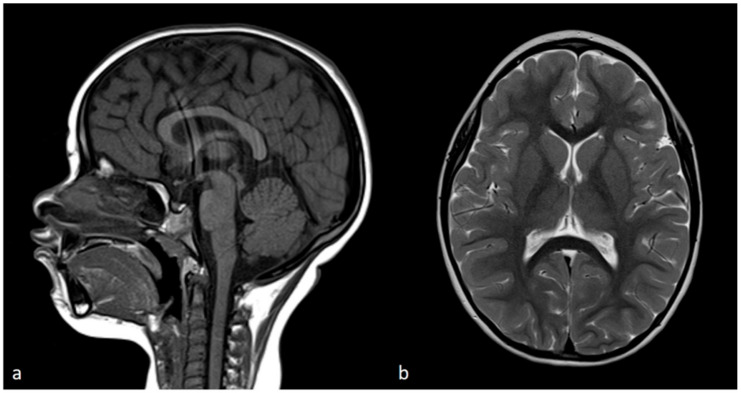
Brain MRI. (**a**) Sagittal T1-weighted image showing complete formation of the corpus callosum and normal shape and volume of cerebellar vermis and brainstem. (**b**) Axial T2-weighted image displays adequate white matter myelinization and normal basal ganglia volumes.

The breakpoint between isodisomy and heterodisomy in 15q was located at chr15:g.60984740 (Table 1, Figure 6 and Figure S2), in a noncoding region between exon 1 and 2 of *RORA* (HGNC:10258) gene (NM_134261.3). *RORA* is associated with intellectual developmental disorder with or without epilepsy or cerebellar ataxia (MIM#618060). There was no deleterious variant identified in *RORA*, but genomic breakpoints can often affect a gene’s function. Both PWS and *RORA* phenotypes are associated with seizures, and it was not clear if any potential abnormality of *RORA* could be contributing to the phenotype in the proband. To account for the possibility of breakpoint affecting the *RORA*’s function, brain magnetic resonance imaging (MRI) was performed. In individuals with a heterozygous *RORA* loss of function, cerebellar hypoplasia and pontocerebellar atrophy are typically indicated. The evaluation of the MRI in the proband was unremarkable (Figure 8). The Sagittal T1-weighted image showed the normal formation of the corpus callosum and the shape and volume of the cerebellar vermis and brainstem. The Axial T2-weighted image displays normal white matter myelinization and basal ganglia volumes.

## 4. Discussion

The prevalence of UPD conditions is reportedly 1 in 3500 [20]. Since the first description of UPD in the etiology of cystic fibrosis in 1988, there have been many studies of the autosomal recessive diseases associated with UPD [21,22,23]. The co-occurrence of PWS with Tay Sachs disease, Bloom syndrome, congenital ichthyosis, and hereditary spastic paraplegia type 11 has been reported [24,25,26,27]. These conditions were associated with UPD, and therefore can also potentially be present in association with Angelman Syndrome (AS), depending on the parental contribution to the isodisomy.

To our knowledge, this is the first report of PWS co-occurrence with calpainopathy. Herein, the patient’s signature characteristics of PWS, comprising of facial dysmorphology, muscular hypotonia, and early poor weight gain followed by severe central early-onset obesity [28,29,30], were molecularly confirmed by the MS-MLPA. However, severe obesity with an earlier onset than expected, persistent muscular hypotonia with elevated CK, and the absence of deep tendon reflexes were atypical for PWS. A trio WGS analysis identified a pathogenic *CAPN3*:c.550del homozygous variant within the isodisomy region.

The *CAPN3* gene is located on 15q15.1–q21.1 [31]. The encoded CAPN3 protein has four domains [32]. Domain IV, the penta-EF-hand (E, E-helix; F, F-helix), is responsible for calcium binding and CAPN3 homodimerization [32]. Domain III, the calpain-type β-sandwich domain, leads the structural changes in the protein upon activation [32]. This domain also enhances CAPN3’s ability to form a homotrimer [33]. Reportedly, homotrimer formation increases the intracellular efficiency of CAPN3 protein within myocytes [33].

*CAPN3*-associated calpainopathy is a rare limb–girdle muscular dystrophy with a prevalence of 1:100,000 [10]. As a member of the calpain family, *CAPN3* has a key role in muscle cell survival, motility, and skeletal plasticity [34,35,36]. Pathogenic variants and the loss of function of *CAPN3* result in the impairment of muscle adaptation and regeneration, and an increase in myonuclear apoptosis [37]. The cumulative muscle atrophy associated with fibrous and adipose tissue hyperplasia leads to calpainopathy. The age range for the onset of symptoms is highly variable [14]. The elevated CK level in young patients is consistent with the early stage of calpainopathy in our patient.

The *CAPN3*:c.550del variant is reportedly a founder variant in European populations [38,39]. Among known *CAPN3* variants, the c.550del is the most prevalent variant in Croatia [39] and has been frequently reported in patients with calpainopathy from countries across Europe [17,38,39,40]. Haplotype analyses have suggested that this variant is rooted in ancestral chromosomes from the ancient Mediterranean population [17,41,42]. The *CAPN3*:550del variant has been reported by several studies [39,42] in patients in the eastern Mediterranean region with suspected calpainopathy. Based on the findings of this study herein, and the prevalence of *CAPN3*:550del, testing for this variant is strongly advised in patients with PWS or AS.

Our trio genome analysis revealed a maternal segmental isodisomy in the PWS region, along with heterodisomy in the more telomeric region of chromosome 15. Complete isodisomy or complete heterodisomy is generally due to trisomy rescue after a chromosome non-disjunction (NDJ) event. NDJ in Meiosis I fails to separate homologous chromosomes and a trisomy rescue event can result in heterodisomy. In contrast, NDJ in Meiosis II fails to separate sister chromatids; therefore, a trisomy rescue event can result in isodisomy. The presence of segmental heterodisomy and isodisomy have been reported in 15q, often due to recombination prior to the NDJ event [43]. After the fertilization of these gametes, postzygotic trisomy rescue preserves the disomy state [44,45]. In segmental UPD cases, homozygosity is often observed in the centromeric region, and heterodisomy in telomeric regions [46].

The breakpoint between maternal isodisomy and heterodisomy was located in the noncoding region of the *RORA* gene. *RORA* is associated with autosomal dominant intellectual developmental disorder with or without epilepsy or cerebellar ataxia (MIM#618060) [47]. The clinical findings of *RORA*-associated disorder have variable phenotypes and include intellectual impairment at varying degrees from normal to severe, speech delay, seizures at multiple types, strabismus, delayed motor development and walking, ataxia, hypotonia, poor coordination and/or mild tremor, hypoplastic cerebellum, and pontocerebellar atrophy [47]. In the proband herein, the phenotypical features expected from *RORA*-associated disorder, namely seizures, developmental delay, speech delay, hypotonia, and broad-based gait overlapped with the PWS or calpainopathy phenotypes. However, abnormal *RORA* is reportedly associated with a distinctive brain MRI pattern of cerebellar hypoplasia and pontocerebellar atrophy in some patients. The MRI was unremarkable in our patient, as shown in Figure 4. Age-related penetrance or phenotypic variability may present confounding factors. Future studies are required to examine the potential functional impact of the breakpoint in the RORA gene.

The American College of Medical Genetics and Genomics has provided a complete set of recommendations for molecular diagnostic testing for UPD [44]. In that article, they list specific chromosome regions for which UPD is implicated. They include paternal UPD6 and transient neonatal diabetes mellitus; maternal UPD7 and Russell–Silver syndrome; paternal UPD11 and Beckwith–Wiedemann syndrome; maternal UPD11 and Russell–Silver syndrome; maternal UPD14 and Temple syndrome; paternal UPD14 and Kagami–Ogata syndrome; maternal UPD15 and Prader–Willi syndrome; Paternal UPD15 and Angelman syndrome; maternal UPD20 and Mulchandani–Bhoj–Conlin syndrome; and paternal UPD20. In these regions, UPD may duplicate an autosomal recessive allele that results in a condition in addition to imprinting. Proper clinical evaluation and genetic counseling is recommended in these cases.

In conclusion, we report the first co-occurrence of PWS and calpainopathy in a patient with a complex phenotype. The joint WGS analysis effectively identified the underlying genetic causes of these unrelated genomic conditions. Despite the rarity of *CAPN3* calpainopathy, but given the high prevalence of *CAPN3*:c.550del variant, the homodisomy of *CAPN3* recessive alleles should be evaluated in patients with unexpected muscle abnormalities with PWS or AS.

## Figures and Tables

**Table 1:** Disomy distribution shows that UPD identified in the proband is segmental isodisomy.

Cytoband	Size	Location	UPD Type
15q11.2–15q22.2	38.61 Mbp	Chr15: 22373341-60984740	Mat Isodisomy
15q22.2–15q26.3	41.55 Mbp	Chr15: 60984741-102531392	Mat Heterodisomy

Chr: Chromosome, UPD: Uniparental Disomy, Mat: Maternal.

## Data Availability

Data for this manuscript are subject to BWH institutional and GDPR privacy policies and restricted from inclusion in repositories. They may be available upon request.

## Supplementary Materials

The following supporting information can be downloaded at: https://www.mdpi.com/article/10.3390/genes15070946/s1, Figure S1: Methylation-specific multiplex ligation-dependent probe amplification (MS-MLPA) of the proband with PWS. The upper panel shows the copy number, and the bottom panel shows the methylation profile. In both panels, each black circle and blue rectangle represent the final probe ratio of the proband and reference sample, respectively. No copy number change was observed in the proband in comparison to the reference sample. Figure S2: The integrative genomics viewer (IGV) of the isodisomy and heterodisomy breakpoint at chr15: g.60984740 in the WGS data. The upper panel shows the mother’s zygosity at chr15: g.60984740 and chr15: g.60984741. The bottom pane shows proband’s zygosity at the same coordinate. Table S1: Zygosity analysis of the variants in 270 OMIM genes located in chromosome 15q.
