# Supplementary figures and images for: Diagnosis of Two Unrelated Syndromes of Prader-Willi and Calpainopathy: Insight from Trio Whole Genome Analysis and Isodisomy Mapping
Source: Genes (Basel). 2024 Jul 19;15(7):946. doi: 10.3390/genes15070946 (PMC11276144; doi:10.3390/genes15070946)

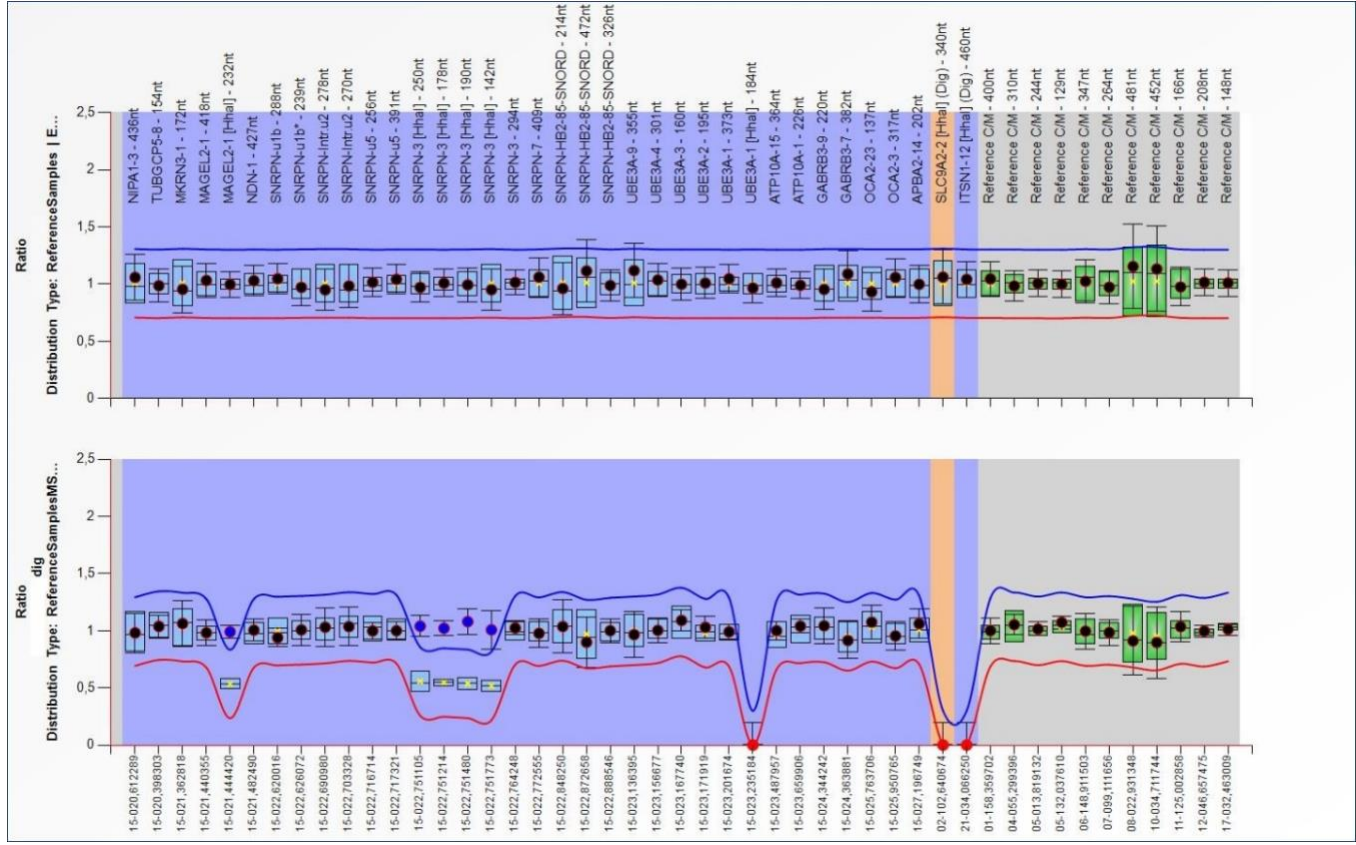

Supplement: Supplementary file 1 [file genes-15-00946-s001.zip › Supplementary Figure S1.pdf]

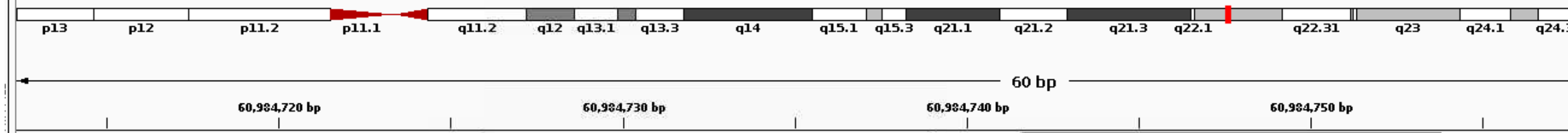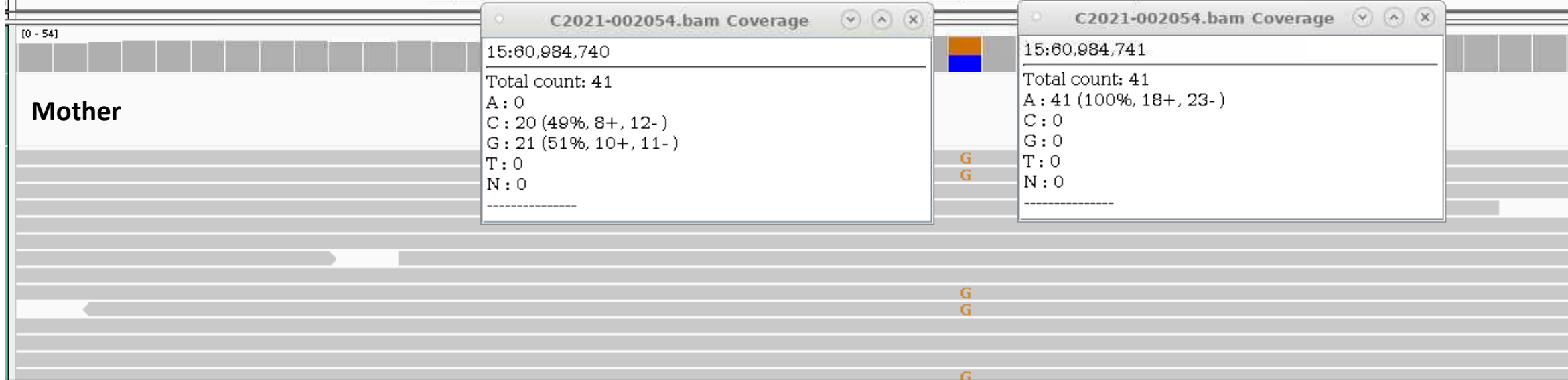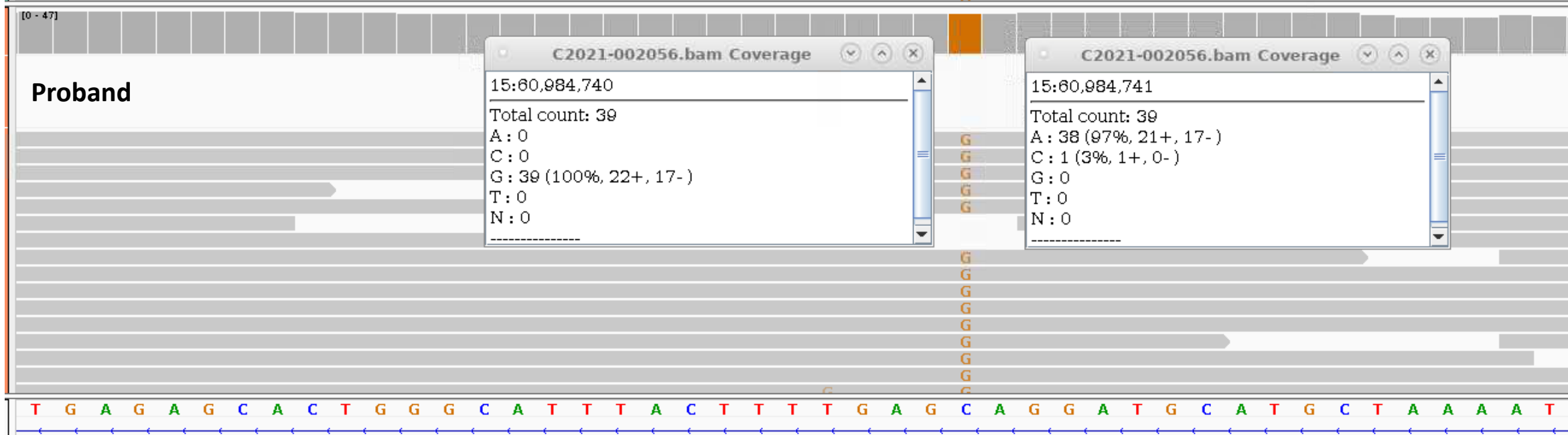

Supplement: Supplementary file 1 [file genes-15-00946-s001.zip › Supplementary Figure S2.pdf]
